# Profiling the immunome of little brown myotis provides a yardstick for measuring the genetic response to white‐nose syndrome

**DOI:** 10.1111/eva.12514

**Published:** 2017-09-03

**Authors:** Michael E. Donaldson, Christina M. Davy, Craig K. R. Willis, Scott McBurney, Allysia Park, Christopher J. Kyle

**Affiliations:** ^1^ Environmental and Life Sciences Graduate Program Trent University Peterborough ON Canada; ^2^ Wildlife Research and Monitoring Section Ontario Ministry of Natural Resources and Forestry Peterborough ON Canada; ^3^ Department of Biology and Centre for Forest Interdisciplinary Research (C‐FIR) University of Winnipeg Winnipeg MB Canada; ^4^ Canadian Wildlife Health Cooperative Atlantic Region Atlantic Veterinary College University of Prince Edward Island Charlottetown PEI Canada; ^5^ Forensic Science Department Trent University Peterborough ON Canada

**Keywords:** genotype‐by‐sequencing, immunogenetics, *Myotis lucifugus*, white‐nose syndrome

## Abstract

White‐nose syndrome (WNS) has devastated populations of hibernating bats in eastern North America, leading to emergency conservation listings for several species including the previously ubiquitous little brown myotis (*Myotis lucifugus*). However, some bat populations near the epicenter of the WNS panzootic appear to be stabilizing after initial precipitous declines, which could reflect a selective immunogenetic sweep. To investigate the hypothesis that WNS exerts significant selection on the immunome of affected bat populations, we developed a novel, high‐throughput sequence capture assay targeting 138 adaptive, intrinsic, and innate immunity genes of putative adaptive significance, as well as their respective regulatory regions (~370 kbp of genomic sequence/individual). We used the assay to explore baseline immunogenetic variation in *M. lucifugus* and to investigate whether particular immune genes/variants are associated with WNS susceptibility. We also used our assay to detect 1,038 putatively neutral single nucleotide polymorphisms and characterize contemporary population structure, providing context for the identification of local immunogenetic adaptation. Sequence capture provided a cost‐effective, “all‐in‐one” assay to test for neutral genetic and immunogenetic structure and revealed fine‐scale, baseline immunogenetic differentiation between sampling sites <600 km apart. We identified functional immunogenetic variants in *M. lucifugus* associated with WNS susceptibility. This study lays the foundations for future investigations of rangewide immunogenetic adaptation to WNS in *M. lucifugus* and provides a blueprint for studies of evolutionary rescue in other host–pathogen systems.

## 1 INTRODUCTION

Host–pathogen dynamics are changing at an unprecedented rate as climate change and human‐mediated transport expand the range of pathogens into previously inhospitable/inaccessible environments (Fisher et al., 2012). As pathogen ranges shift, disease‐related population declines in naïve wildlife populations often threaten population persistence, as evidenced by several emerging wildlife diseases (Gallana, Ryser‐Degiorgis, Wahli, & Segner, 2013; Smith et al., 2012). Selective forces exerted by infectious diseases can rapidly influence the distribution of adaptive genetic variants associated with disease susceptibility over short timescales (Gallana et al., 2013). For infectious diseases of conservation significance, this process of local adaptation can result in evolutionary rescue of a population, where disease‐resistant animals survive a strong selective sweep from disease and pass their resistance to their offspring (Carlson, Cunningham, & Westley, 2016; Maslo & Fefferman, 2015). Spatial patterns of local adaptation to strong selective sweeps may be linked to particular gene variants favored in local interactions (Hansen, Olivieri, Waller, & Nielsen, 2012; Kyle et al., 2014; Rico, Morris‐Pocock, Zigouris, Nocera, & Kyle, 2015; Schoville et al., 2012). Determining how these variants are spread or localized among populations is essential to understanding and managing the emergence of new selective pressures, such as emerging infectious diseases (Eizaguirre, Lenz, Kalbe, & Milinski, 2012; Kyle et al., 2014).

White‐nose syndrome (WNS) is a recently emerged disease in hibernating bats caused by the fungal pathogen *Pseudogymnoascus destructans*. The fungus was introduced from Eurasia to North America, where it was first documented in Schoharie County, New York, in 2006 (Blehert et al., 2009; Leopardi, Blake, & Puechmaille, 2015). WNS has spread rapidly across North America, causing >80% declines in some eastern bat populations (Frick et al., 2010, 2015; Langwig et al., 2012; Lorch et al., 2016). While several North America bats are highly susceptible to WNS, European bats do not experience mortality from infection with *P. destructans* (Puechmaille, Fuller, & Teeling, 2011; Puechmaille, Wibbelt, et al., 2011). Controlled experiments with captive bats show that identical strains of *P. destructans* cause mortality in North American little brown myotis (*Myotis lucifugus*) but not in European greater mouse‐eared bats (*Myotis myotis*; Davy et al., 2017), suggesting a genetic basis for immunotolerance or immunoprotection.

There are several promising leads for the development of treatments for WNS (e.g., Cheng et al., 2016; Cornelison, Gabriel, Barlament, & Crow, 2014; Wilcox & Willis, 2016), but no effective mitigation or treatment protocols are currently available. However, some populations near the epicenter of WNS may be stabilizing following their initial, precipitous declines (Dobony et al., 2011; Langwig et al., 2012, 2017). Persistence of these populations does not seem to be associated with immigration (Maslo, Valent, Gumbs, & Frick, 2015), but may indicate evolution of resistance or tolerance to the disease (Langwig et al., 2017). Thus, adaptation and evolutionary rescue may be the best hope for recovery of bat populations affected by WNS (Maslo & Fefferman, 2015). Understanding patterns of immunogenetic adaptation to WNS is therefore critical to determining disease management strategies and recovery programs for the affected populations.

Immune genes mediate the initial response of individuals to pathogens and in many cases, the acquisition of immunity. At the population level, genetic diversity of immune genes influences resistance or tolerance to disease via pathogen‐mediated balancing selection (Eizaguirre et al., 2012; Rico et al., 2015). Studies of wildlife populations generally focus on adaptive immunity, which has often been assessed by using genetic diversity in the major histocompatibility complex (MHC). Diversity at the MHC provides a proxy for potential to adapt to shifting pathogen pressures, due to the role of MHC in pathogen recognition and pathogen susceptibility (Acevedo‐Whitehouse & Cunningham, 2006; Eizaguirre et al., 2012; Kyle et al., 2014). Some studies of immunogenetic diversity also include receptor genes associated with innate immunity (e.g., Toll‐like receptors and interleukins) and these markers have revealed spatial patterns of resistance to emerging infectious diseases such as chytridiomycosis and mycoplasmosis (Bonneaud, Balenger, Zhang, Edwards, & Hill, 2012; Savage & Zamudio, 2011).

The MHC Drb1 locus in *M. lucifugus* is among the most polymorphic recorded in mammals to date (Palmer et al., 2016). Pyrosequencing of 160 individuals sampled across Canada suggests that balancing selection has maintained similar MHC diversity among genetically differentiated subpopulations, which may be disrupted by WNS‐mediated immunogenetic selection (Davy et al., 2017). However, the extreme observed polymorphism of the Drb1 locus in *M. lucifugus* is due partly to multiple gene duplications, which limits the use of these data. Furthermore, susceptible bats infected with *P. destructans* upregulate multiple, complementary immune responses (Field et al., 2015; Lilley et al., 2017; Moore et al., 2013; Rapin et al., 2014), so immunogenetic selection by WNS cannot be fully captured by experimental designs that target single, candidate genes. No other population‐level immunogenetic analyses exist for *M. lucifugus,* or for any other North American species of bats threatened by WNS. Fortunately, new molecular tools allow more comprehensive investigation of immunogenetic adaptation (Harrisson, Pavlova, Telonis‐Scott, & Sunnucks, 2014).

Genotype‐by‐sequencing (GBS) assays have emerged as a cost‐effective method for obtaining population‐level assessments of neutral and functional genetic variation, and identifying local adaptation (Tiffin & Ross‐Ibarra, 2014). GBS assays involve enriching for genomic subsets of DNA via restriction enzyme‐, amplicon‐, or hybridization‐based methods (Jones & Good, 2016), conducting high‐throughput sequencing and identifying single nucleotide polymorphisms (SNPs). Targeted approaches, including amplicon‐ and hybridization‐based GBS, have been used in wildlife studies to identify SNPs in specific coding and regulatory regions of immune genes, collectively called the “immunome.” Targeted GBS can identify population‐level immunogenetic shifts in response to pathogens, and has been applied to a range of species, including the Tasmanian devil (*Sarcophilus harrisii*; Morris, Wright, Grueber, Hogg, & Belov, 2015), turkey (*Meleagris gallopavo*; Reed, Mendoza, & Settlage, 2016), gray wolf (*Canis lupus*; Schweizer et al., 2016), thinhorn sheep (*Ovis dalli*; Roffler et al., 2016), and red fox (*Vulpes vulpes*; Donaldson et al., unpublished). GBS is an attractive option for understanding the impacts of WNS on immunogenetic diversity in bat populations, because it allows accurate characterization of diversity at duplicated loci, and cost‐effective targeting of multiple, relevant genes. Regardless of the genomic coverage of high‐throughput sequencing methods, population genetic analyses still rely on adequate sample sizes to detect the genetic signature of selection by pathogens or other selective pressures, reinforcing the importance of a cost‐effective approach.

We developed a novel hybridization‐based GBS assay to characterize the genetic diversity of the *M. lucifugus* immunome. Our assay includes 170 loci, including 120 immune genes and their regulatory areas, 18 Drb1‐like exon 2 regions, and 32 neutral loci to allow characterization of neutral population structure, against which hypotheses of local adaptation can be tested. We applied this assay to test the hypothesis that WNS exerts significant selection on the immunome of affected bat populations. Controlling for neutral genetic population structure, we predicted immunogenetic divergence would be detectable between WNS‐naïve populations and populations affected by WNS. This study provides a foundation for future investigations of rangewide immunogenetic adaptation to WNS in *M. lucifugus* and other affected species of bats.

## 2 MATERIALS AND METHODS

### 2.1 Microsatellite markers, immune genes and probe development

We developed our assay for primary application to *M. lucifugus* because this species' genome is publicly available (Myoluc2.0 genome assembly, Ensembl release version 81; Cunningham et al., 2015), and recent research has identified putative “WNS‐response” genes for this species (Rapin et al., 2014), which informed our selection of target genes for sequence capture.

To assess functional immunogenetic variation, we assembled a list of 120 candidate genes related to immune system processes based on (i) the Human Innate & Adaptive Immune Responses RT^2^ Profiler PCR Array (Qiagen); (ii) a review of innate and adaptive immunity, development, and signaling (Knight, 2013); (iii) a study of gene expression in *M. lucifugus* following infection with *P. destructans* (Rapin et al., 2014); and (iv) a gene ontology (GO) term search in the *M. lucifugus* Ensembl database for GO records related to fungi (including cellular response to molecule of fungal origin, defense response to fungus, and neutrophil‐mediated killing of fungus). We used this candidate gene list to query the *M. lucifugus* Myoluc2.0 genome assembly and created a BED‐formatted file containing coordinates for all exons. Additionally, we targeted potential regulatory regions by including coordinates for the 1,500‐bp region upstream from the first exon for each gene. Finally, we added exon 2 coordinates for 18 Drb1‐like genes identified in Ensembl that putatively encode functional full‐length proteins.

To target putative neutral markers for the detection of genetic population structure, we selected 32 microsatellite markers for *M. lucifugus* from the published literature (Burns, Broders, & Frasier, 2012; Castella & Ruedi, 2000; Johnson et al., 2014; Oyler‐McCance & Fike, 2011; Piaggio, Figueroa, & Perkins, 2009; Trujillo & Amelon, 2009). Using these primer sets, we added coordinates for these markers to the BED‐formatted file. In total, the final BED‐formatted file contained coordinates for 170 loci. Descriptions for protein‐coding and microsatellite regions are provided in the Supporting Information (Tables S1–S2).

Custom NimbleGen SeqCap EZ probes (Roche) were produced for “primary targets” using the BED‐formatted file and the *M. lucifugus* Myoluc2.0 genome assembly as a reference. We added 100‐bp “padding” to each target to increase the efficiency of the sequence capture, and we used a “relaxed” probe design that allowed up to 20 close matches to the *M. lucifugus* reference genome. We compared our probes to the *M. lucifugus* reference genome to ensure that our assay had a low likelihood for “off‐target” sequence capture: 91% of the probes matched only their target sequence, and 99% had five or fewer matches to the *M. lucifugus* reference genome.

### 2.2 Sample collection, DNA extraction, and quantification

All work was conducted under approved animal care protocols from the University of Winnipeg and the Ontario Ministry of Natural Resources and Forestry. To test the relative impacts of geographic location and exposure to *P. destructans* on neutral and immunogenetic population structure in *M. lucifugus*, we assigned bat samples collected in eastern Canada to three post hoc groups (Table S3). The first group included bats that were nonharmfully sampled at a hibernaculum in Manitoba, Canada (MB, *n* = 28), that did not contain *P. destructans* at the time of sampling. The second group contained bats from two hibernacula near Thunder Bay, Ontario, which were also sampled before the arrival of *P. destructans* (ON, *n* = 36). Wing biopsies from these bats were immediately stored in RNA*later* (Qiagen) following sampling. These two groups represent our “pre‐WNS treatment.” The third group came from populations of bats in Atlantic Canada that had been exposed to WNS for at least one winter, but were found moribund or dead in the winter of 2014 in the Atlantic provinces of Nova Scotia and Prince Edward Island (ATL, “post‐WNS treatment,” *n* = 28). These bats were submitted to the Canadian Wildlife Health Cooperative (CWHC), Atlantic Region for necropsy. Of these post‐WNS bats, 15 were diagnosed as positive for WNS, 12 were diagnosed as suspect D for WNS, and 1 was negative for WNS using the approved diagnostic categories for WNS found in the Canadian Bat WNS Necropsy Protocol (CWHC, 2014), and they were assumed to not be immunotolerant nor immunocompetent to WNS. Wing tissue was collected from the left dactylopatagium major during these necropsies and stored in lysis buffer (4 M urea, 0.2 M NaCl, 0.5% n‐lauroyl sarcosine, 10 mM 1,2‐cyclohexanediaminetetraacetic acid, 0.1 mM Tris–HCl pH 8.0) until analysis. We dissolved all tissue samples in lysis buffer containing 600 U/ml proteinase *K* at 56°C for 2 hr. We extracted DNA using either the automated 96‐well MagneSil Blood Genomic Max Yield System (Promega) or the DNeasy Blood and Tissue Kit (Qiagen). We then quantified all DNA extractions using the Quant‐iTPicoGreen dsDNA Assay Kit (ThermoFisher Scientific).

To investigate the possibility that our assay could also be used to investigate immunogenetic variation and adaptation in other species affected by WNS, we also isolated DNA from “post‐WNS” *Eptesicus fuscus* (*n* = 2) from New Brunswick, both suspect B for WNS (CWHC, 2014) and *M. septentrionalis* (*n* = 2) from Nova Scotia and Prince Edward Island, positive and suspect D for WNS, respectively (CWHC, 2014), and included these samples in the assay.

### 2.3 DNA library preparation, sequence capture, and high‐throughput sequencing

We prepared DNA libraries using the KAPA HTP Lib Prep Kit (Roche) and performed the sequence capture using the NimbleGen SeqCap EZ Developer Library kit v5.1 (Roche) with the following modifications to the manufacturer's protocol. Each DNA library preparation used 150 ng total DNA. TruSeq HT Dual‐Index Adapters (Integrated DNA Technologies) resuspended in Nuclease Free Duplex Buffer (Integrated DNA technologies) were used at a final concentration of 600 nM instead of the SeqCap Adapter Kits A and B (Roche) during adapter ligation. We performed 11 cycles during the LM‐PCR, and initial DNA library quality was assessed by ethidium bromide‐stained gel electrophoresis using a 2% E‐Gel (ThermoFisher Scientific). We used 1 μl of the xGen Universal Blocking Oligo TS HT‐i5 (Integrated DNA Technologies) and 1 μl xGen Universal Blocking Oligo TS HT‐i7 (Integrated DNA Technologies) instead of the NimbleGen Multiplex Hybridization Enhancing Oligo Pool (Roche), and we used NimbleGen SeqCap EZ Developer Reagent (Roche) instead of NimbleGen COT Human DNA (Roche) during hybridization sample preparation. The hybridization was carried out at 47°C for 72 hr. We assessed the pooled target‐enriched DNA quality using a bioanalyzer (Agilent Technologies) and performed high‐throughput sequencing on a HiSeq 2500 rapid run using 2 × 100‐bp reads on a single flow cell (Illumina).

### 2.4 Sequence alignment, variant annotation, and SNP/INDEL analysis

We used the bwa‐mem command in the burrows‐wheeler aligner v0.7.12 (bwa; Li, 2013) to align paired‐end reads to the Myoluc2.0 genome sequence and compiled sequence alignment metrics using samtools v1.2 (Li et al., 2009). We used the genome analysis toolkit v3.5 (gatk; McKenna et al., 2010) for base quality score recalibration, realignment of insertions/deletions (INDELs), duplicate removal, depth of coverage calculations, SNP/INDEL discovery, and genotyping across all samples, using standard hard filtering parameters or variant quality score recalibration according to gatk best practices recommendations (DePristo et al., 2011; Van der Auwera et al., 2013).

### 2.5 Analysis of targeted microsatellites

We used two different approaches to assign microsatellite genotypes. The first method (gatk) relied on sequence alignment to the *M. lucifugus* genome. We identified a single INDEL to represent each microsatellite by selecting the short tandem repeat that yielded the highest: (i) percentage of heterozygotes; (ii) gatk “quality” score; or (iii) number of alleles. For each of these three scenarios, we used the gatk to calculate the number of heterozygotes for each marker using a subset of our data that included only the 36 ON samples and the 28 MB samples. These 64 samples were previously genotyped based on traditional PCR amplification and sequencing of 11 microsatellite markers (Davy et al., 2017). We calculated the number of heterozygotes for each of these markers, to assess whether our sequence capture assay could be used to build on previous microsatellite‐based studies. Our second genotyping method used the Galaxy platform (Afgan et al., 2016) to run STR‐FM (Galaxy Version 1.0.0; Fungtammasan et al., 2015) and identify di‐ and tetra‐nucleotide STRs from the raw Illumina.fastq data, without genome alignment.

### 2.6 Analysis of functional loci and identification of novel, putatively neutral SNPs

We used gatk to assemble a master variant call format file (.vcf) that included SNPs with a maximum missing genotype frequency of 5% and a minimum minor allele frequency of 2%. We then used gatk to generate subdatasets of SNPs from specific categories (exon, intron, regulatory region, and Drb1‐like exon 2). For the “off‐target” SNPs, we used the Ensembl variant effect predictor tool to determine the bp distance between a SNP and the closest gene and generated a list of putatively neutral SNPs that were at least 100,000 bp from a gene (e.g., Kawakami et al., 2014), which we considered to be in linkage disequilibrium. We “binned” these SNPs based on the minor allele frequency, and tested for genetic structure (see below) using the SNPs with minor allele frequency values of 2% and 25%. All.vcf files were reformatted using pgdspider v2.0.9.2 (Lischer & Excoffier, 2012) for downstream analyses.

To explore variation in functional regions, we ran two lositan analyses (Antao, Lopes, Lopes, Beja‐Pereira, & Luikart, 2008; Beaumont & Nichols, 1996) to identify F_ST_ outliers that are putatively under selection. lositan parameters included 1,000,000 iterations, a 99.5% confidence interval, a false discovery rate (FDR) threshold of 0.05, and a stepwise mutation model. We enabled the “Neutral mean F_ST_” and the “Force mean F_ST_” options. The first analysis used population priors based on geography (MB, ON, and ATL) and the second considered exposure to WNS (pre‐WNS, post‐WNS). We extracted the subset of directional F_ST_ outliers identified in each analysis with vcftools v0.1.14 (Danecek et al., 2011) and used them to explore immunogenetic population structure (see below).

SNPs that alter amino acids or affect splicing regions can have major effects on the function of encoded proteins. We considered F_ST_ outliers that had these particular consequences as the most likely signals of either local adaptation to pre‐occurring pathogens (in the geographic comparison) or alleles disproportionately selected against by WNS (in the pre‐ and post‐WNS comparison). Mutations in regulatory regions can also influence gene expression and ultimately affect disease outcome (Fraser, 2013) so we also identified SNPs within regulatory regions, although the functional results of these mutations cannot be inferred from sequence capture data alone.

### 2.7 Characterization of neutral and immunogenetic population structure

We used two a priori groupings to test for neutral genetic and immunogenetic population structure: (i) geographic grouping (MN, ON, and ATL), or (ii) grouping by exposure to WNS (pre‐ and post‐WNS). We explored genetic structure based on the different SNP datasets using structure v2.3.4 (Pritchard, Stephens, & Donnelly, 2000) and parallelized the runs using the strauto v0.3.1 script (Chhatre & Emerson, 2017). We ran structure with a burn‐in length of 50,000 followed by 200,000 iterations for *K* = 1 through 4, and each run was performed 20 times. We used structure harvester web v0.6.94 (Earl & VonHoldt, 2012) to calculate the ΔK statistic (Evanno, Regnaut, & Goudet, 2005). Multiple structure runs were combined with clumpp v1.1.2 (Jakobsson & Rosenberg, 2007) using the Greedy option (10,000 repeats), and we visualized the results using distruct v1.1 (Rosenberg, 2004). We also performed principal component analysis (PCA) using adegenet v2.0.0 (Jombart & Ahmed, 2011). We obtained the required “genlight” objects for the adegenet analysis using a combination of vcftools and plink v1.07 (Purcell et al., 2007) to reformat the.vcf files to plink‐formatted files (.raw).

## 3 RESULTS

### 3.1 High‐throughput sequencing, sequence alignment, and depth of coverage

**Figure 1:**
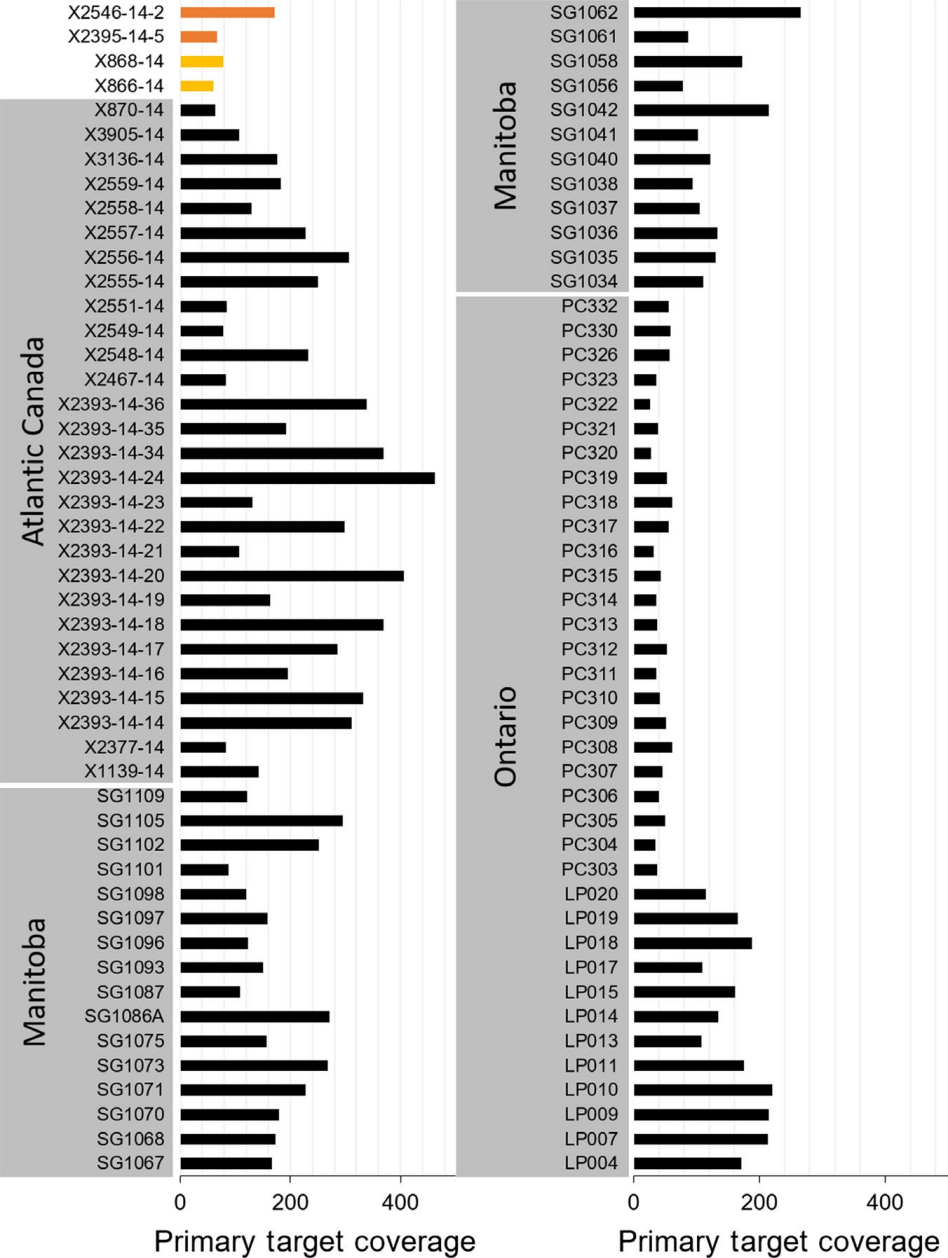
Mean depth of coverage for target loci, sorted by sample ID and sampling location. Black bars represent the primary target species, *Myotis lucifugus*. Orange bars: *M. septentrionali*s; yellow bars: *Eptesicus fuscus*

**Figure 2:**
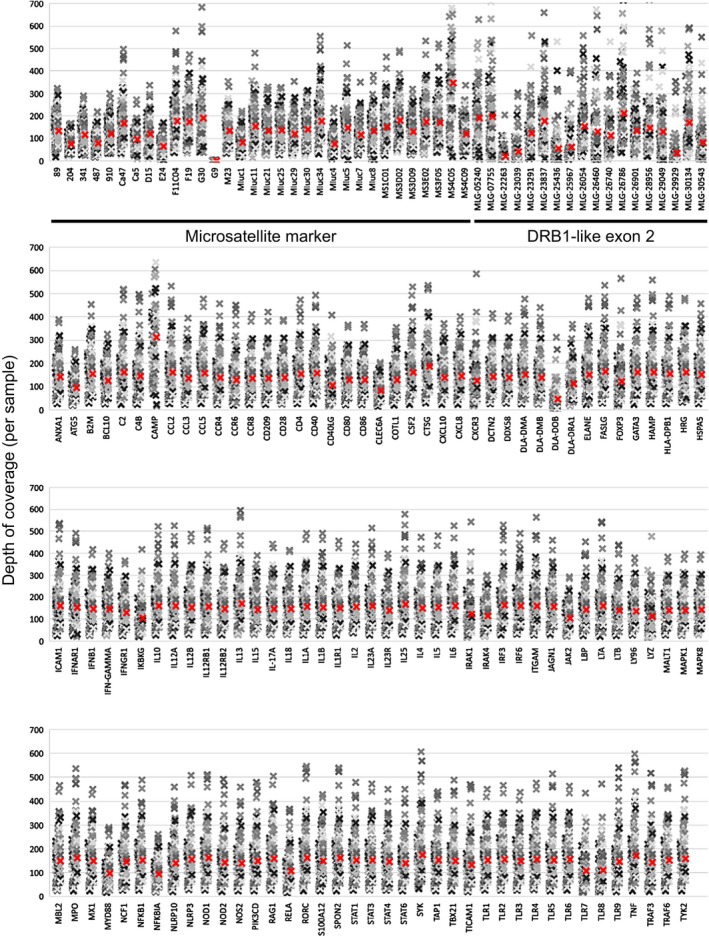
Primary target depth of coverage per sample, sorted by locus. Mean depth of coverage for each locus is indicated by a red colored “x.” Microsatellite markers and Drb1‐like exon 2 targets are marked in the top panel. We condensed the presented data by abbreviating Ensembl‐derived *Myotis lucifugus* gene (MLG) identifiers, where “MLG‐”=“ENSMLUG000000,” for the Drb1‐like genes that did not have informative Ensembl or GenBank gene names

NimbleGen sequence capture and high‐throughput sequencing yielded 717 million paired‐end reads for 96 libraries. We mapped 712 million of these reads to the *M. lucifugus* genome (Table S4). Sequencing alignment and depth of coverage metrics (Tables 1 and 2) indicated that the *M. lucifugus* probes were successful in capturing the targeted loci in *E. fuscus* and *M. septentrionalis*. Primary target enrichment was 42.1%, 41.4%, and 37.3% for *M. lucifugus, E. fuscus,* and *M. septentrionalis*, respectively (Table 1) and coverage for sequenced samples from *E. fuscus* (59.5X, 78.2X) and *M. septentrionalis* (66.5X, 171.0X) fell within the observed range for *M. lucifugus* (26.2X–463.3X; Table 2). To visualize the variation in depth of coverage across samples and the primary targets, we plotted the mean depth of coverage for primary targets across all samples (Figure 1) and the depth of coverage obtained from each sample for the primary targets (Figure 2). Overall, we determined average coverage was high for microsatellite markers (135X), Drb1‐like exon 2 targets (121X), and targeted immune genes (145X).

**Table 1:** High‐throughput sequencing and read alignment summary statistics

Sample category	Total mapped reads	Total mapped reads filtered (%)	Duplicates (%)	Mapping quality (%)	Multimapped reads (%)	Total mapped reads [pass filter]	Reads mapped to primary targets [pass filter]	Primary target enrichment [pass filter] (%)
*Myotis lucifugus* (*n* = 92)								
Mean	7,459,167	80.39	73.70	6.60	0.09	1,411,920	656,230	42.1
Minimum	4,203,530	54.45	47.70	5.95	0.06	524,146	118,002	15.5
Maximum	11,483,159	91.95	85.37	9.43	0.14	3,061,461	2,023,112	66.3
*Eptesicus fuscus* (*n* = 2)								
Minimum	3,935,930	83.44	67.23	15.58	0.22	651,945	277,998	40.2
Maximum	6,080,356	84.98	69.17	15.98	0.23	913,531	367,312	42.6
*M. septentrionalis* (*n* = 2)								
Minimum	6,548,133	80.67	70.80	9.62	0.19	913,750	300,166	32.8
Maximum	9,467,611	86.05	76.22	9.68	0.21	1,830,053	765,227	41.8

**Table 2:** Primary target depth of coverage summary

Sample category	*N*	Mean	Minimum	Maximum
*Myotis lucifugus* (total)	92	148.1	26.2	463.3
Ontario (Hibernaculum 1)	12	164.9	108.2	220.9
Ontario (Hibernaculum 2)	24	44.4	26.2	61.1
Manitoba	28	159.8	78.1	294.6
Atlantic Canada	28	218.2	62.9	463.3
*Eptesicus fuscus*	2	–	59.5	78.2
*M. septentrionalis*	2	–	66.5	171.0

### 3.2 Microsatellite genotyping via INDEL detection

When processing the 32 microsatellite loci included in our assay, gatk analysis identified 400 INDELs for the 32 loci, demonstrating that unique microsatellite regions contained multiple INDEL calls. However, the relatively short read length obtained with our sequencing method failed to reliably capture entire short tandem repeat (STR) regions. Thus, microsatellite genotypes could not be recovered for all samples. As a result, heterozygous genotypes scored from our high‐throughput sequencing differed from the previous results obtained using traditional PCR methods (Davy et al., 2017) by −25% to −42%. The STR‐FM analysis, which does not rely on aligning reads to the genome, was also unable to generate genotypes for more than two microsatellite markers using a subset of our samples (data not shown). Therefore, we did not conduct further analyses with the microsatellite data.

### 3.3 Analysis of neutral genetic structure

**Figure 3:**
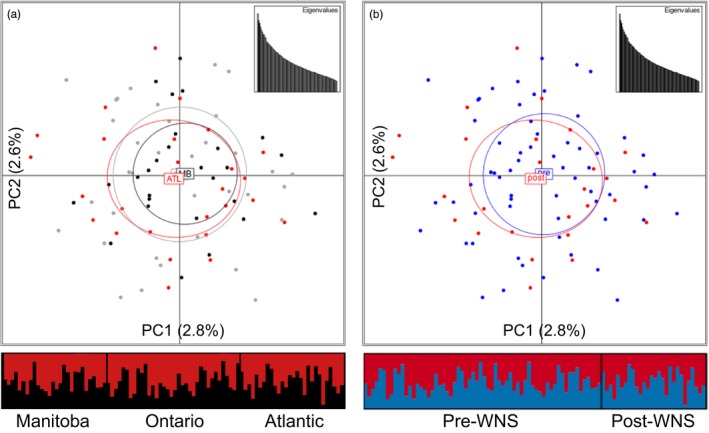
Visualizing lack of genetic structure using 1,038 putatively neutral SNPs (>100 kpb from nearest gene) with max‐missing genotype of 5% and minor allele frequency of 2% for *Myotis lucifugus* (*n* = 92). Samples were grouped based on (a) geographic location or (b) previous exposure to WNS. Principal component analysis plots were produced using adegenet, and the percentage of variation for each axis and a scatter plot of eigenvalues are included for each analysis; barplot shows results of structure analysis (*K* = 2). MB = Manitoba (black); ON = Ontario (gray); ATL/post = Atlantic Canada/post‐WNS (red); pre = pre‐WNS (blue)

The gatk analysis identified 16,115 “off‐target” SNPs. The Ensembl variant effect predictor tool found that 1,038 of these SNPs were located >100,000 bp from a neighboring gene (Table 3; Table S5). The putatively neutral SNPs map to 111 different clusters (>100,000 bp from the next cluster) on 88 different scaffolds of the *M. lucifugus* genome sequence assembly. We found no evidence for genetic structure based on these putatively neutral SNPs in the adegenet‐ or structure‐derived plots based on geography (Figure 3a), or based on the presence of WNS in those areas (Figure 3b), regardless of the minor allele frequency cutoff used in the analysis (data not shown).

**Table 3:** Summary of the number of detected, putatively neutral single nucleotide polymorphisms (SNPs) binned by minor allele frequency (MAF)

MAF (%)	SNPs (nr)
2	1038 (111)
5	544 (90)
10	343 (77)
15	236 (68)
20	169 (58)
25	142 (53)

nr = “nonredundant” number of neutral SNP clusters with long‐distance (>100‐Kbp) SNPs.

### 3.4 Immunome SNP detection and analyses

We identified 17,495 SNPs within the primary target loci, located in exons (3,536 SNPs), introns (5,482 SNPs), and regulatory regions (5,482 SNPs). lositan identified 328 and 299 directional outlier SNPs in the geography‐ and WNS‐based analyses, respectively, 32 of which were detected in both analyses. We acknowledge that false positives for SNPs under selection are common in outliers detected using F_ST_‐based methods (Narum & Hess 2011); therefore, the candidate SNPs identified in this experiment will require further validation in future studies. The predicted impacts of each of those 595 directional outlier SNPs are summarized in Tables 5 and S6. Focusing on SNPs most likely to cause major functional changes, we found that 23 outlier SNPs in 19 genes in the geographic comparison resulted in an amino acid change, as did 28 SNPs in 21 genes in the WNS comparison (Table 6). In the WNS comparison, an outlier SNP in the intron region of HLA‐DPB1 resulted in likely modification of the splice donor sequence. We also detected 194 SNPS in the regulatory regions of 78 genes (Table S6), of which 11 were identified in both comparisons (Table 4).

**Table 4:** Summary of lositan F_ST_ outliers (FDR < 0.05) in targeted immunome features of *Myotis lucifugus* (*n* = 92). Directional outliers were used for structure and adegenet analyses (Figure 4)

Feature type	Geography		WNS	
	Directional outliers (nr)	Balancing outliers (nr)	Directional outliers (nr)	Balancing outliers (nr)
Exon	60 (35)	89 (45)	72 (43)	416 (94)
Intron	159 (62)	249 (71)	131 (54)	1,050 (99)
Regulatory Region	109 (54)	141 (74)	96 (50)	666 (116)
Total	328 (151)	479 (190)	299 (147)	2,132 (309)

nr = “nonredundant” number of genes with F_ST_ outliers.

**Figure 4:**
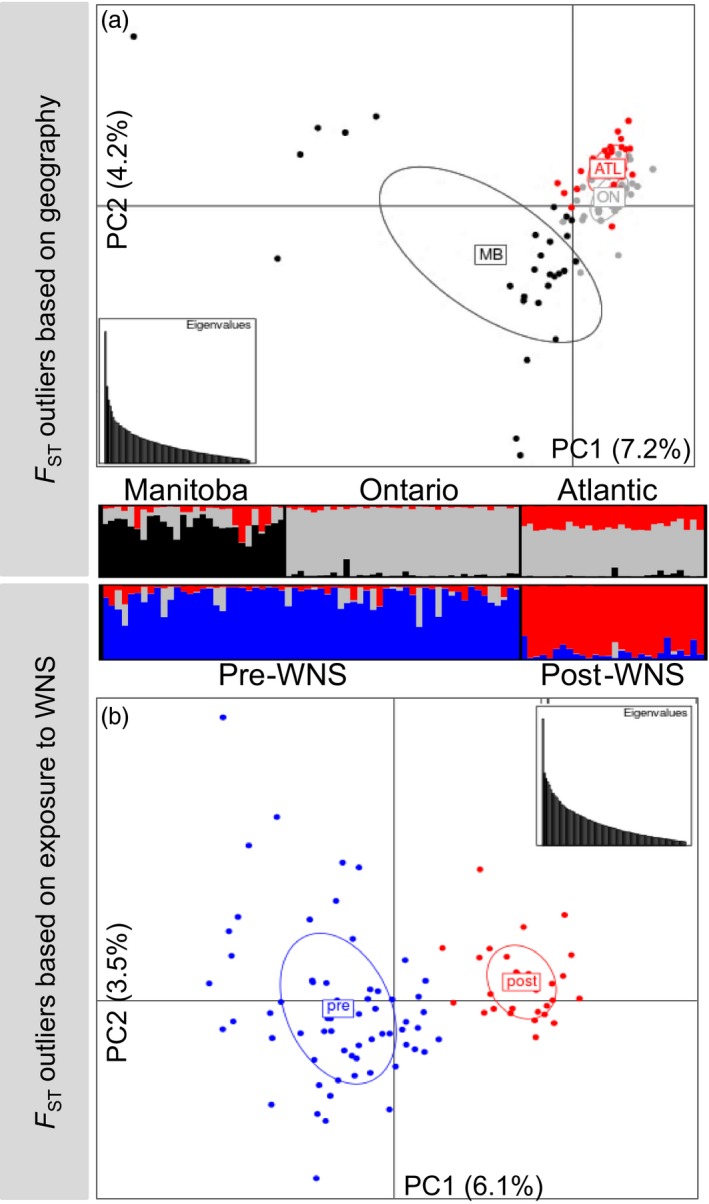
Analysis of immunogenetic population structure based on lositan‐detected F_ST_ outliers with max‐missing genotype of 5% and minor allele frequency of 2% for *Myotis lucifugus* (*n* = 92). Samples were grouped based on (a) geographic location or (b) previous exposure to WNS. Principal component analysis plots were produced using adegenet, and the percentage of variation for each axis and a scatter plot of eigenvalues are included for each analysis; barplot shows results of structure analysis (*K* = 3). MB = Manitoba (black); ON = Ontario (gray); ATL/post = Atlantic Canada/post‐WNS (red); pre = pre‐WNS (blue)

Analyses of genetic structure in adegenet and structure did not identify geography‐ or WNS‐associated genetic structure using the entire primary target locus, exon, intron, or regulatory region SNP datasets (data not shown). However, using the lositan‐predicted outlier SNPs, we observed subtle immunogenetic structure based on geography or the presence of a brief period of co‐occurrence with *P. destructans* (Figure 4). The analysis based on geography identified immunogenetic differentiation between *M. lucifugus* in MB and conspecifics in ON and ATL (Figure 4a), while analysis based on co‐occurrence with *P. destructans* grouped ON and MB together (pre‐WNS), differentiated from the post‐WNS samples from ATL (Figure 4b).

## 4 DISCUSSION

### 4.1 Sequence capture and high‐throughput sequencing

Reduced representation genomic profiling strategies have emerged as valuable alternatives to whole‐genome sequencing (Narum, Buerkle, Davey, Miller, & Hohenlohe, 2013) where population‐level assessments are not yet feasible for nonmodel organisms with larger genomes. Reduced representation approaches can include both transcriptome studies (all expressed genes) or GBS that can include restriction site association DNA (RAD) marker, target capture, and amplicon sequencing. While RAD sequencing has many advantages when genomic resources for the species are sparse, it has many limitations in identifying patterns of local adaptation (Andrews, Good, Miller, Luikart, & Hohenlohe, 2016). Amplicon sequencing of a large number of loci has many advantages in elucidating the genetic variation from known targets; however, in this instance, we chose a sequence capture approach to also pull down large segments of the immunome that included upstream regulatory regions of the genes of interest. As such, the candidate gene GBS approach employed in this study provided several advantages over other means in obtaining immunogenetic information that is likely to be influenced by the selective pressures from disease such as that caused by *P. destructans* (Table 6; Figure 4).

Here, we found target enrichment led to even sequencing uniformity/coverage, which has been noted by other research groups (Powell, Amish, Haynes, Luikart, & Latch, 2016; Samorodnitsky et al., 2015; Schweizer et al., 2016). The assay we developed provided a high on target means to obtain moderate to high coverage of each target (26–463X; Table 1) that was relatively even across samples and loci (Figures 1, 2). The assay also worked across other species (*M. septentrionalis* and *E. fuscus*) that are known to also be impacted by WNS to varying degrees (Frank et al., 2014; Frick et al., 2015; Langwig et al., 2012). As such, this assay sets the stage for cross‐species analyses to further our understanding of the variable immune responses to this disease. The assay, however, yielded far too high a percentage of duplicates (54%–92%; Table 1) that compromised the level of coverage. The duplicates were likely a matter of too many PCR cycles at the adapter ligation stage during DNA library preparations. In the future, we would decrease from 11 to 6–8 cycles in the LM‐PCR step. One aspect of the assay that did not meet expectations was the amplification of microsatellite loci, largely as a matter of the sequencing technology used (HiSeq 2500 rapid run using 2 × 100‐bp reads on a single flow cell). The hybridization‐based method we employed to capture microsatellite loci was successful; however, we were unable to generate genotypes using the reads containing short tandem repeats. To avoid these experimental design and technical problems, we recommend using sequencing technologies that offer longer read lengths. The Illumina MiSeq and Life Technologies PGM System currently yield 300‐bp to 400‐bp read lengths, which may allow for the microsatellite and flanking regions to be sequenced, and should help microsatellite analysis in nonmodel organisms. Overall, the assay provided a relatively high number of variable neutral SNPs with frequency differences amenable to population genetic analyses (Table 3) and a large number of F_ST_ outlier SNPs in exons, introns, and regulatory regions (Table 4), several of which were predicted to have important variant effects (Table 5).

**Table 5:** Summary of consequences predicted by the variant effect predictor, for directional F_ST_ outliers detected by lositan from immunome sequence capture of *Myotis lucifugus* samples (*n* = 92)

Feature	Consequence	Geography	WNS
Exon	Synonymous variant	36	43
Exon	Missense variant	21	26
Exon	Missense variant, splice region variant	2	2
Exon	Splice region variant, synonymous variant	1	1
Intron	Intron variant	157	122
Intron	Splice region variant, intron variant	2	8
Intron	Splice donor variant	0	1
Regulatory Region	Upstream gene variant	109	96

### 4.2 Immunogenetic diversity and structure

We developed a GBS sequence capture assay to cost‐effectively and rapidly reveal genetic diversity in the immunome of endangered *M. lucifugus* threatened by mass die‐offs from WNS. The assay characterized neutral population structure to control for stochastic immunogenetic differentiation among sampled areas (Table S5; Figure 3), and also elucidated genetic variation and structure of immune genes via hundreds of SNPs within the exons, introns, and regulatory regions of those genes (Table S6; Figure 4). Preliminary application of the assay to *E. fuscus* and *M. septentrionalis* indicates it may also be an effective tool for these species. By targeting the assay to address specific research questions, our GBS approach can be used across the range of *M. lucifugus* to investigate drivers of genetic, morphological, and behavioral variation.

Our assay revealed subtle immunogenetic variation and structure on a relatively small geographic scale, suggestive of local immunogenetic adaptation within an otherwise panmictic population (Figures 3a and 4a; Davy et al., 2017). Comparisons of samples taken before and after the arrival of WNS suggest a nonrandom removal of genetic variants in the immunome by *P. destructans* (Figures 3b and 4b). If similar selection is occurring in *M. lucifugus* that are surviving in WNS‐impacted areas, there may be potential for rapid local adaptation to WNS, raising the possibility of evolutionary rescue (Carlson et al., 2016; Maslo & Fefferman, 2015). Conversely, immunogenetic selection by WNS may disrupt previously adaptive patterns of immunogenetic variation as *P. destructans* continues to spread, further complicating the recovery of *M. lucifugus*. Our interpretation of this data is effected by the possibility that the 28 *M. lucifugus* we sampled from Atlantic Canada were not exposed to *P. destructans* during the previous year, and while 27 of these individuals died of WNS in 2014, this might have been their first exposure to an infection with *P. destructans*. To partially address this concern, we note 14 of 28 *M. lucifugus* with sample IDs “X2393‐14‐N” (where N varies; Table S3) came from a hibernaculum in Prince Edward Island where WNS mortality was identified in the previous winter, 1 year prior to these individuals dying of WNS and being collected for this study.

Immunogenetic diversity in *M. lucifugus* is extremely high. Previous attempts to quantify variation were complicated by duplication of loci in the MHC of *M. lucifugus*, which exhibits up to 24 Drb1‐like loci (Davy et al., 2017; Palmer et al., 2016). Our targeted sequence capture assay controls for this gene duplication and allows genotypes to be unambiguously assigned to each individual. We detected functionally significant differentiation in several Drb1‐like loci associated with both geography and previous exposure to WNS (Table 6). Exposure to WNS is also associated with a shift in genetic variation at interleukins and Toll‐like receptors (Davy et al., 2017; Field et al., 2015; Lilley et al., 2017; Rapin et al., 2014), consistent with the hypothesis that WNS exerts immunogenetic selective pressure on *M. lucifugus*. Our research on the interactions between *M. lucifugus* genetics and *P. destructans* continues to reinforce the need to take both interindividual and inter‐regional variation of both the host and pathogen into account when interpreting genetic data. In this study, bats collected from sites <600 km apart in Manitoba and Ontario belong to a panmictic population based on neutral molecular markers, but exhibit local variation in the immunome that may result in different expression of immune genes among sites (Table 6). For example, it is possible that local immunogenetic differentiation between these sites result in different survival rates following the introduction of WNS. Variation in the regulatory regions (Table S6) could also alter the expression of integral immune genes among sites.

**Table 6:** lositan‐detected F_ST_ outliers from SNP analyses based on a priori grouping by geographic location (Manitoba, Ontario, and Atlantic) or by WNS exposure history (pre‐WNS, post‐WNS). Only the SNPs that are most likely to have a functional impact by altering amino acids or affecting splice sequences are listed (see Methods and Table S6 for details)

Comparison (#SNPs)	Gene name	Ensembl transcript ID	Brief description	Amino acids
Geographic	CCL3	ENSMLUT00000002888	C‐C motif chemokine	A/V
Geographic	CCR4	ENSMLUT00000027956	Chemokine (C‐C motif) receptor 4	S/F
Geographic	CD40	ENSMLUT00000006008	CD40 molecule, TNF receptor superfamily member 5	S/N
Geographic	Drb1e2‐like‐e	ENSMLUT00000027881	DLA class II histocompatibility antigen	N/H
Geographic	Drb1e2‐like‐f	ENSMLUT00000028450	DLA class II histocompatibility antigen	T/M
Geographic	Drb1e2‐like‐l	ENSMLUT00000029278	DLA class II histocompatibility antigen	L/R
Geographic	Drb1e2‐like‐n	ENSMLUT00000030076	DLA class II histocompatibility antigen	E/D
Geographic	Drb1e2‐like‐r	ENSMLUT00000027745	DLA class II histocompatibility antigen	Q/L^a^
Geographic (2)	HRG	ENSMLUT00000013351	Histidine‐rich glycoprotein	K/R, H/Q
Geographic	IFNGR1	ENSMLUT00000008611	Interferon gamma receptor 1	D/E
Geographic (2)	IL12RB1	ENSMLUT00000013802	Interleukin 12 receptor, beta 1	K/R, T/I
Geographic (2)	IL1R1	ENSMLUT00000011035	Interleukin 1 receptor, type I	R/K, E/K
Geographic	IL23A	ENSMLUT00000006770	Interleukin 23, alpha subunit p19	R/T
Geographic	IRF6	ENSMLUT00000004509	Interferon regulatory factor 6	K/N
Geographic (2)	MPO	ENSMLUT00000006099	Myeloperoxidase	Q/L^a^, G/R
Geographic	NLRP10	ENSMLUT00000000818	NLR family, pyrin domain containing 10	S/C
Geographic	NOS2	ENSMLUT00000015896	Nitric oxide synthase	G/D
Geographic	RAG1	ENSMLUT00000000542	Recombination activating gene 1	S/N
Geographic	SPON2	ENSMLUT00000017687	Spondin 2, extracellular matrix protein	T/M
WNS	CCR4	ENSMLUT00000027956	Chemokine (C‐C motif) receptor 4	I/N
WNS	DDX58	ENSMLUT00000003044	DEAD (Asp‐Glu‐Ala‐Asp) box polypeptide 58	V/I
WNS	DLA‐DRA1	ENSMLUT00000027968	DLA class II histocompatibility antigen, DR alpha chain‐like	P/T
WNS	Drb1e2‐like‐i	ENSMLUT00000031273	DLA class II histocompatibility antigen	E/V
WNS (2)	Drb1e2‐like‐k	ENSMLUT00000023434	DLA class II histocompatibility antigen	D/N, D/E
WNS	Drb1e2‐like‐p	ENSMLUT00000022698	DLA class II histocompatibility antigen	S/N
WNS (2)	Drb1e2‐like‐r	ENSMLUT00000027745	DLA class II histocompatibility antigen	Q/L^a^, R/H
WNS	HLA‐DPB1	ENSMLUT00000016285	Major histocompatibility complex, class II, DP beta 1	b
WNS	IFNAR1	ENSMLUT00000025403	Interferon (alpha, beta and omega) receptor 1	S/P
WNS	IL12RB1	ENSMLUT00000013802	Interleukin 12 receptor, beta 1	I/L
WNS	IL12RB2	ENSMLUT00000001415	Interleukin 12 receptor, beta 2	I/V
WNS (2)	IL1R1	ENSMLUT00000011035	Interleukin 1 receptor, type I	L/M, D/G
WNS	IL5	ENSMLUT00000016553	Interleukin 5	K/E
WNS (2)	ITGAM	ENSMLUT00000011332	Integrin, alpha X (complement component 3 receptor 4 subunit)	Q/R, V/L
WNS	MPO	ENSMLUT00000006099	Myeloperoxidase	Q/L^a^
WNS (3)	NOD2	ENSMLUT00000015164	Nucleotide‐binding oligomerization domain containing 2	L/V, S/R, S/A
WNS	NOS2	ENSMLUT00000015896	Nitric oxide synthase	A/V
WNS	TBX21	ENSMLUT00000014543	T‐box 21	Q/P
WNS	TLR1	ENSMLUT00000008406	Toll‐like receptor 1	V/I
WNS	TLR2	ENSMLUT00000012815	Toll‐like receptor 2	S/P
WNS (2)	TLR6	ENSMLUT00000008414	Toll‐like receptor 6	H/L, I/V
WNS	TLR9	ENSMLUT00000015105	Toll‐like receptor 9	A/V

a Indicates outlier SNPs were identified in both the geographic and WNS‐based comparisons.

b Indicates a SNP predicted to have a high impact by altering a splice donor sequence in an intron. The other SNPs listed here are in exons and are predicted have moderate impacts by altering the amino acid sequence.

High immunogenetic variation in *M. lucifugus* has implications for the interpretation of gene expression studies as well. Bats from different sampling sites may respond differently to immune challenges due to variation in exon and regulatory regions of the immunome. Therefore, experimental gene expression studies related to *P. destructans* or other pathogens should explicitly control for potential geographic variation. Otherwise, observed differences in gene expression cannot be unambiguously attributed to the effects of the pathogen (or other treatments of interest).

The GBS approach used here provides a basis for real‐time investigations of evolutionary rescue in populations of bats that persist following initial declines from WNS (Maslo & Fefferman, 2015). The results of our study were based on analysis of a small number of populations, and in the post‐WNS population, the *M. lucifugus* we examined did not survive infection. Ideally, future studies will compare immunogenetic variation not only among exposed and unexposed sites, but also among time‐series samples taken from bats that have survived multiple selective sweeps from one, two, or more winters in hibernacula containing *P. destructans*.

## 5 CONCLUSION

We developed a cost‐effective high‐throughput sequence capture assay to test for immunogenetic shifts in *M. lucifugus* populations following exposure to *P. destructans*. Sequence analysis from 92 *M. lucifugus* identified sequence variation in 138 immune‐related genes, their upstream regulatory regions, and 111 putatively neutral regions of the genome. The “one‐pot” assay we developed to test for genetic structure and immunogenetic variation identified functional immunogenetic variants in *M. lucifugus* putatively associated with WNS susceptibility, demonstrated a shift in immunogenetic diversity of populations pre‐ and post‐WNS exposure, and provided preliminary support for a potential evolutionary rescue of *M. lucifugus* in Atlantic Canada given a nonrandom purging of immunogenetic variants in the WNS‐susceptible bats. We can use the genetic variants identified in this study as a baseline for future investigations of rangewide immunogenetic adaptation to WNS in little brown myotis. Ultimately, understanding the potential for evolutionary rescue in a species can guide more effective and targeted management actions to mitigate the impacts of WNS on North American bat populations. Overall, this study sets the stage for further research with larger sample sizes and increased population replicates under different types of selective pressure to further understand patterns of local adaptation in this bat species, most importantly in context of WNS exposure and survival.

## CONFLICTS OF INTEREST

The authors declare no conflict of interest.

## AUTHOR CONTRIBUTIONS

MED, CMD, CKRW, and CJK conceived and designed the experiments. SM, JS, and CKRW collected bat tissue. SM performed postmortem diagnosis of WNS. MED performed the experiments and analyzed the data. CJK contributed reagents/materials/analysis tools. MED, CMD, CKRW, CJK, and SM wrote and revised the manuscript.

## DATA ARCHIVING STATEMENT

All high‐throughput sequencing.fastq files have been archived in the NCBI Sequence Read Archive database (accession number SRP100885). The *M. lucifugus* SNP data file (.vcf) is available on the Dyrad Digital Repository (https://doi.org/10.5061/dryad.2r4c7).
