# Supplementary material for: Profiling the immunome of little brown myotis provides a yardstick for measuring the genetic response to white‐nose syndrome
Source: Evol Appl. 2017 Sep 3;10(10):1076–90. doi: 10.1111/eva.12514 (PMC5680615; doi:10.1111/eva.12514)
Supplement: Supplementary file 1 [file EVA-10-1076-s001.pdf]

Supplementary Material

Tables S1-S4. Frequency of hybrids recorded in samples of natural mosquito populations from published material. African countries are classified as belonging to the Western or Eastern region according to their location relative to the Great Rift Valley system, which represents a major geographical barrier structuring *An. gambiae sensu lato* populations. The number of surveys refers essentially to the number of localities where collections were carried out; in some instances, however, the same locality was surveyed more than once in different years or seasons. The total number of studies is not the sum of the number of studies referred to individual countries because a few studies covered more than one country.

Table S1. Frequency of *Anopheles arabiensis* × *gambiae* hybrids in samples of adult female mosquitoes from continental Africa.

Region	Country	No. Hybrids (%)		No. Specimens	No. Surveys	No. Studies
West Africa	Burkina Faso	3	(0.026%)	11,337	498	9
	Cameroon	0	(0.000%)	3,287	155	3
	Gambia	1	(0.009%)	10,595	53	3
	Ghana	0	(0.000%)	924	4	2
	Guinea Bissau	0	(0.000%)	87	1	1
	Guinea Conakry	0	(0.000%)	156	1	1
	Mali	3	(0.016%)	18,415	97	6
	Nigeria	6	(0.040%)	15,000	82	7
	Senegal	0	(0.000%)	56,525	36	11
	Total West Africa	13	(0.011%)	116,354	689	43
East Africa	Burundi	1	(0.152%)	658	1	1
	Kenya	1	(0.009%)	11,027	45	11
	Madagascar	0	(0.000%)	530	14	4
	Mozambique	0	(0.000%)	184	6	1
	Sudan	0	(0.000%)	277	3	1
	Tanzania	2	(0.035%)	5,664	10	2
	Zambia	0	(0.000%)	203	17	1
	Zimbabwe	0	(0.000%)	107	1	1
	Total East Africa	4	(0.022%)	18,650	97	22
Grand Total		17	(0.015%)	135,004	786	65

Table S2. Frequency of *Anopheles coluzzii* × *gambiae s.s.* hybrids in samples of adult female mosquitoes from West and Central Africa.

Region	Country	No. Hybrids (%)		No. Specimens	No. Surveys	No. Studies
Central Africa	Angola	0	(0.00%)	499	5	3
	Cameroon	0	(0.00%)	2,577	61	7
	Central African Republic	0	(0.00%)	91	1	1
	Congo	1	(1.92%)	52	1	1
	Democratic Republic of Congo	0	(0.00%)	47	1	1
	Equatorial Guinea	0	(0.00%)	108	2	2
	Total Central Africa	1	(0.03%)	3,374	71	15
West Africa	Benin	1	(0.50%)	202	5	2
	Burkina Faso	44	(0.66%)	6,653	267	13
	Ghana	4	(0.21%)	1,868	19	7
	Guinea Conakry	2	(0.39%)	516	4	2
	Ivory Coast	1	(0.44%)	226	7	2
	Mali	1	(0.08%)	1,205	14	6
	Niger	0	(0.00%)	18	1	1
	Nigeria	0	(0.00%)	2,432	11	2
	Total West Africa	53	(0.40%)	13,120	328	35
High Hybridization Area (HHA)	Gambia	44	(2.46%)	1,789	24	3
	Guinea Bissau	37	(21.51%)	172	2	1
	Senegal	25	(2.11%)	1,185	10	3
	Total HHA	106	(3.37%)	3,146	36	7
	Grand Total	160	(0.81%)	19,640	435	57

Table S3. Frequency of *Anopheles arabiensis* × *gambiae* hybrids in larval samples from continental Africa.

Region	Country	No. Hybrids (%)		No. Specimens	No. Surveys	No. Studies
West Africa	Angola	0	(0.00%)	22	1	1
	Gambia	0	(0.00%)	563	6	3
	Mali	0	(0.00%)	1,034	1	1
	Nigeria	0	(0.00%)	3,075	13	4
	Total West Africa	0	(0.00%)	8,211	22	10
East Africa	Kenya	0	(0.00%)	9,315	31	11
	Total East Africa	0	(0.00%)	9,315	31	11
Grand Total		0	(0.00%)	17,526	53	21

Table S4. Frequency of *Anopheles coluzzii* × *gambiae s.s.* hybrids in larval samples from West Africa.

Country	No. Hybrids (%)		No. Specimens	No. Surveys	No. Studies
Ghana	0	(0.00%)	237	2	2
Mali	4	(0.57%)	701	1	1
Nigeria	0	(0.00%)	264	6	1
Total	4	(0.33%)	1,202	9	4
