# Supplementary material for: Profiling the immunome of little brown myotis provides a yardstick for measuring the genetic response to white‐nose syndrome
Source: Evol Appl. 2017 Sep 3;10(10):1076–90. doi: 10.1111/eva.12514 (PMC5680615; doi:10.1111/eva.12514)
Supplement: Supplementary file 2 [file EVA-10-1076-s002.pdf]

Supporting Table S5. Frequency of homospecific and heterospecific insemination in *Anopheles gambiae s.l.* taxa from Burkina Faso.

		**Female**					**Homospecific Mating (%)**
		arabiensis	*coluzzii*	*gambiae s.s.*	*arabiensis* x *coluzzii*	Total	
**Sperm**	*arabiensis*	317	1	5	1	324	97.8
	coluzzii	3	409	16	1	429	95.3
	gambiae s.s.	2	18	217	1	238	91.2
	absent	38	14	23	0	75	
	Total	360	442	261	3	1 066	
**Insemination (%)**		89.4	96.8	91.2	100.0	93.0	
