# Supplementary material for: Profiling the immunome of little brown myotis provides a yardstick for measuring the genetic response to white‐nose syndrome
Source: Evol Appl. 2017 Sep 3;10(10):1076–90. doi: 10.1111/eva.12514 (PMC5680615; doi:10.1111/eva.12514)
Supplement: Supplementary file 3 [file EVA-10-1076-s003.pdf]

Supporting Table S6. Analysis of Deviance of generalized linear models testing the effect of frequency-dependent hybridization in *An. arabiensis*, *An. coluzzii*, and *An. gambiae* s.s., based on the strength of their pair sexual isolation indices I_{PSI}. The parameter estimates of the minimal adequate model (No. 1, containing all main effects and their interaction) are given in Table 2.

Model Terms	Residual Deviance	AIC	Change in Deviance	d.f.	F-test	*P*
1. Frequency × Taxa	0.001131	–48.492				
2. Frequency + Taxa	0.008556	–32.258	0.007425	2	13.128	0.017
3. Taxa	0.016756	–27.537	0.008200	1	5.750	0.053
4. Frequency	0.028610	–24.187	0.020054	2	7.031	0.027
5. Null Model	0.029052	–26.034	0.020496	3	4.791	0.049
