# Supplementary material for: Profiling the immunome of little brown myotis provides a yardstick for measuring the genetic response to white‐nose syndrome
Source: Evol Appl. 2017 Sep 3;10(10):1076–90. doi: 10.1111/eva.12514 (PMC5680615; doi:10.1111/eva.12514)
Supplement: Supplementary file 4 [file EVA-10-1076-s004.pdf]

Supporting Table S7. Frequency of *Anopheles coluzzii* × *gambiae s.s.* hybrids in larval samples of populations from the rainforest eco-climatic domain of Cameroon, Central Africa.

**Locality (altitude) / Period**	**No. Hybrids (%)**		**No. *An. coluzzii***	**No. *An. gambiae***	**No. Surveys**
		Guinean side of CVL			
Ekondo Titi Beach (10 m) – Dec 2012	0	(0.00%)	79	4	1
Ekondo Titi Town (45 m) – Dec 2012	0	(0.00%)	6	112	1
Idenau (8 m) – Oct 2012	0	(0.00%)	26	0	1
Kumba (210-263 m) – Mar 2010	0	(0.00%)	4	182	1
Mamfe (49-94 m) – Mar 2010	0	(0.00%)	14	159	1
Nguti (230 m) – Mar 2010	0	(0.00%)	1	86	1
Total Guinean	0	(0.00%)	130	543	6
		Cameroon Volcanic Line (CVL)			
Buea (480-790 m) – Mar 2010	2	(0.97%)	75	129	1
Loum (218-263 m) – Apr 2010	0	(0.00%)	45	9	1
Mbanga (116-123 m) – Apr 2010	0	(0.00%)	67	77	1
Njombe (88-94 m) – Apr 2010	0	(0.00%)	36	1	1
Nkongsamba (800-924 m) – Apr 2010	0	(0.00%)	134	8	1
Tibati (850-880 m) – Aug 2012	1	(0.22%)	12	433	1
Tibati (850-880 m) – Sep 2012	2	(0.20%)	23	962	1
Tibati (850-880 m) – Oct 2012	1	(0.44%)	3	221	1
Total CVL	6	(0.27%)	395	1,840	8
		Congolese side of CVL			
Bouandjo (4 m) – Jun 2010	0	(0.00%)	34	19	1
Bwambe (8-24 m) – Jun 2010	0	(0.00%)	60	61	1
Campo (0-20 m) – Jun 2010	0	(0.00%)	157	121	1
Douala (0-50 m) – Mar 2010	0	(0.00%)	123	8	1
Ebodje (0-4 m) – Jun 2010	0	(0.00%)	239	12	1
Eboundja (27 m) – Jun 2010	0	(0.00%)	1	75	1
Grand Batanga (10-23) – Jun 2010	0	(0.00%)	73	15	1
Kribi (4-20 m) – Jun 2010	0	(0.00%)	359	52	1
Lolabe (0 m) – Jun 2010	0	(0.00%)	30	66	1
Nziou (4-20 m) – Jun 2010	0	(0.00%)	251	25	1
Tiko (8-49 m) – Mar 2010	0	(0.00%)	116	4	1
Yaoundé (700-760 m) – May 2008	0	(0.00%)	173	130	14
Yaoundé (700-760 m) – Jun 2008	0	(0.00%)	139	72	12
Yaoundé (700-760 m) – Jul 2008	1	(0.36%)	175	98	13
Yaoundé (700-760 m) – Aug 2008	1	(0.55%)	108	73	12
Yaoundé (700-760 m) – Sep 2008	1	(0.32%)	243	72	15
Yaoundé (700-760 m) – Oct 2008	1	(0.20%)	369	134	15
Yaoundé (700-760 m) – Nov 2008	0	(0.00%)	201	20	11
Yaoundé (700-760 m) – Dec 2008	0	(0.00%)	185	30	14
Yaoundé (700-760 m) – Jan 2009	0	(0.00%)	196	43	10
Yaoundé (700-760 m) – Feb 2009	0	(0.00%)	277	120	12
Yaoundé (700-760 m) – Mar 2009	0	(0.00%)	114	46	9
Yaoundé (700-760 m) – Apr 2009	0	(0.00%)	194	70	12
Yoyo – Dec 2012	0	(0.00%)	78	0	1
Total Congolese	4	(0.08%)	3,895	1,366	160
**Grand Total**	10	(0.12%)	4,420	3,749	174
